# Integrating Weight Bias Awareness and Mental Health Promotion Into Obesity Prevention Delivery: A Public Health Pilot Study

**DOI:** 10.5888/pcd10.120185

**Published:** 2013-04-04

**Authors:** Gail L. McVey, Kathryn S. Walker, Joanne Beyers, Heather L. Harrison, Sari W. Simkins, Shelly Russell-Mayhew

**Affiliations:** Author Affiliations^:^ Kathryn S. Walker, Heather L. Harrison, The Hospital for Sick Children, Toronto, Ontario, Canada; Joanne Beyers, Sudbury and District Health Unit, Sudbury, Ontario, Canada; Sari W. Simkins, Toronto, Ontario, Canada; S. Russell-Mayhew, University of Calgary, Alberta, Canada.

## Abstract

**Introduction:**

Promoting healthy weight is a top priority in Canada. Recent federal guidelines call for sustained, multisectoral partnerships that address childhood obesity on multiple levels. Current healthy weight messaging does not fully acknowledge the influence of social determinants of health on weight.

**Methods:**

An interactive workshop was developed and implemented by a team of academic researchers and health promoters from the psychology and public health disciplines to raise awareness about 1) weight bias and its negative effect on health, 2) ways to balance healthy weight messaging to prevent the triggering of weight and shape preoccupation, and 3) the incorporation of mental health promotion into healthy weight messaging. We conducted a full-day workshop with 342 Ontario public health promoters and administered a survey at preintervention, postintervention, and follow-up.

**Results:**

Participation in the full-day workshop led to significant decreases in antifat attitudes and the internalization of media stereotypes and to significant increases in self-efficacy to address weight bias. Participants reported that the training heightened their awareness of their own personal weight biases and the need to broaden their scope of healthy weight promotion to include mental health promotion. There was consensus that additional sessions are warranted to help translate knowledge into action. Buy-in and resource support at the organizational level was also seen as pivotal.

**Conclusion:**

Professional development training in the area of weight bias awareness is associated with decreases in antifat attitudes and the internalization of media stereotypes around thinness. Health promoters’ healthy weight messaging was improved by learning to avoid messages that trigger weight and shape preoccupation or unhealthful eating practices among children and youth. Participants also learned ways to integrate mental health promotion and resiliency-building into daily practice.

## Introduction

Approximately one-third of children both in Canada and the United States are overweight or obese (1,2). Nutrition and physical activity promotion alone may not be an effective obesity prevention strategy (3). The promotion of life skills such as stress management, assertive communication, and social problem-solving offer complementary self-care options that can easily be incorporated into mainstream obesity prevention work (4–6). Health promoters in chronic disease prevention receive little or no training in these areas. Moreover, the rates of weight bias among educators and health professionals exceed rates in the general population (7,8). Feeling stigmatized triggers emotional and physical symptoms of stress and undermines the adoption of health-promoting behaviors (9). Overweight or obese youth may continue to face the negative consequences of prejudice unless changes are made to the societal factors that reinforce weight stigma (10). For this reason, practice recommendations have been developed to incorporate weight bias awareness into obesity prevention work (11).

An interdisciplinary team of researchers and health promoters collaborated to plan, develop, implement, and evaluate a professional development model entitled LENS (Leveraging Equitable Non-Stigmatizing health promotion delivery). Given the pervasiveness of weight bias among health professionals (7,8) and its direct influence on discourses about weight, nutrition, and physical activity, the professional development aimed to reduce the antifat attitudes of the health promoters. The intervention provided an overview of weight bias awareness as well as the multiple ecological influences that affect health, such as mental well-being, social and economic factors, and the built environment. Developers also emphasized skill building in mental health promotion and ways to deliver healthy weight messaging without triggering weight and shape preoccupation. The primary purpose of the pilot study was to evaluate whether or not the intervention led to changes in the participants’ antifat attitudes, internalization of media stereotypes, body satisfaction, and sense of self-efficacy to address weight bias. A second goal was to determine the overall satisfaction with and feasibility of participating in the workshop, and lessons learned about the role of mental health promotion in the promotion of healthy weights.

## Methods

Participants were 342 public health practitioners (94% female) working in provincially funded public health units across Ontario, Canada (12). Participants were drawn from 3 health units whose work focused on nutrition, chronic disease prevention (healthy eating, physical activity, tobacco-free living, cancer screening), and injury prevention. The practitioners’ work mandate is to promote and protect the health of Canadians through leadership, partnership, innovation, and action in public health (12). The mean number of years spent in public health practice was 10.75 years (standard deviation [SD], 9.5 years; range, 0-37 years). In their daily practice, 22.8% indicated they work currently with infants; 60% work with children, 66% work with youth, 74% work with adults, and 48% work with families. All practitioners participated in the intervention, and 325 consented to be part of the research study (95% response rate), which involved the completion of self-report measures. A subsample of 42 participants, randomly selected from the 325, agreed to take part in an additional brief semistructured interview several weeks after the workshop.

A prospective design without randomization or involvement of a control group was used (13) because the goal of the pilot was to assess whether the expected goals of the training were met and to provide some evidence to support the continuation of the model in future studies (14).

### Measures

Self-reported information was collected from participants about their sex, ethnocultural background, and the number of years spent working in public health. The Antifat Attitudes Questionnaire (15) was used as an extrinsic measure of weight bias. This 13-item scale measures peoples’ negative feelings toward fat people, personal concern about becoming fat, and the belief that being overweight is a matter of personal responsibility or lack thereof. Items are scored on a 9-point Likert-type response scale anchored from 0, “very strongly disagree” to 9, “very strongly agree.” Participants respond to the questions with a number on the scale. Cronbach’s ɑ for this measure is 0.80. Higher scores indicate higher levels of antifat attitudes and weight bias.

The internalization subscale (5 items) of the Sociocultural Attitudes Towards Appearance Questionnaire (SATAQ) was used to measure the degree of internalization of sociocultural stereotypes of weight and appearance (16). Scores range from 8 to 40; higher scores represent a greater internalization of media-depicted images of the thin ideal. A 5-point scale (1, definitely disagree; 2, mostly disagree; 3, neither agree nor disagree; 4, mostly agree; 5, definitely agree) is used. This scale has established validity (16). The ɑ coefficient for this measure is 0.91.

Body satisfaction was measured by using the 6-item version of the Body Satisfaction Scale (17). Participants rate how happy they are with 6 different body areas/attributes on a 5-point scale, where 1 was completely unhappy, 2 was somewhat unhappy, 3 was neither happy nor unhappy, 4 was happy, and 5 was completely happy. Total scores are calculated; higher scores represent greater body satisfaction. The ɑ coefficient for this measure is 0.88.

The Self-Efficacy to Change weight-related social norms was used to examine participants’ self-efficacy to change other people’s attitudes and behaviors concerning food and weight (18). The scale consists of 7 items ranked on a 4-point Likert scale (4 = definitely, 3 = maybe, 2 = probably not, 1 = definitely not). Higher scores reflect greater self-efficacy to change behavior. The ɑ coefficient for the scale is 0.65.

We developed a 12-item open-ended self-report survey to solicit feedback from participants about their overall satisfaction with the intervention and its feasibility in terms of fit and utility with their daily practice. Survey items included the following questions: What did you like the most about today’s session? Is there anything you would change? What is your opinion about the material presented? Are there other materials you think would be helpful to include in the workshop? How do you feel about filling in the questionnaires? Was the length of the intervention adequate in terms of preparing you to learn this new information? How feasible was it to attend a day-long workshop? Would you see value in having follow-up sessions conducted with you on a one-on-one basis? Based on what you learned today, what are 3 immediate next steps you could put into practice either in your work life or personal life?

A semistructured interview administered by telephone 6 weeks after the workshop detailing critical incidents or short descriptions of meaningful events (19) was used to solicit information about ways in which the workshop led to changes, if any, at home or at work. In adult education, critical incidents allow people to respond to a set of specific prompts aimed at uncovering their learning experiences (20). Questions probe participants’ responses in domains of behavior, affect, and cognition. The rationale for critical incidents in this study was to measure the meaningful reaction of participants (20).

The professional development workshop, designed to improve professional practice in health promotion (not treatment) with the general public (not with referred clients), centered around 3 themes (Box): 1) weight bias awareness (21–24); 2) a balanced approach to healthful eating and healthy weight messaging (25,26); and 3) mental health promotion (4–6,27,28). The training consisted of a group-based, day-long workshop that was facilitated by psychologists with expertise in professional development in mental health promotion and eating disorder prevention. The workshop objectives were achieved by way of didactic research sharing, multimedia components, personal reflection activities, and collaborative group conceptualization activities.

### Procedure

Members of our interdisciplinary team, who came from academic settings, public health units, eating disorder prevention units, and provincial resource centers that focused on nutrition, physical activity, or chronic disease/cancer prevention, planned extensively to build the intervention. This team met for 1 year to collaborate on the development of the research questions, the intervention and its alignment with current workplace mandates, and plans for its eventual strategic dissemination across multiple sectors. Ethics approval was obtained from the Research Ethics Board at The Hospital for Sick Children and at 3 public health units in Ontario, Canada. Participants were then contacted by e-mail through their managers about the study and invited to participate. Information about the study, which the researchers provided, was circulated via the collaborating public health managers. Professional development workshops were delivered from April 2010 through January 2011. The workshop intervention was carried out in groups of 50 (approximately 7 in total), on a first come, first serve basis. After giving written consent, participants were asked to complete a self-report survey on 3 occasions: 1) immediately before the intervention, 2) immediately after the day-long professional development intervention, and 3) six weeks later. The first 2 surveys were completed privately in a group-based setting in the room where the intervention was held. With participant consent, the third survey was sent individually to each participant by e-mail 6 weeks after their participation in the workshop. Most study participants (98%) returned their postworkshop survey immediately following the intervention (n = 318), and 65% returned their 6-week follow-up survey (n = 208). All were given the opportunity to participate in the workshop regardless of study consent. Members of the research team conducted in-depth, semistructured telephone interviews lasting 20 to 25 minutes at the 6-week follow-up with a subsample (n = 42) of the participants who consented to be contacted.

### Statistical analyses

For the self-report survey data, a mixed-model repeated measures analysis of covariance (ANCOVA) was conducted on each of the 4 outcome variables with time (baseline, postworkshop, and 6-week follow-up) as the within-subject variable and baseline scores as the covariate. The analysis was significant for all of the 4 outcome variables. Open-ended self-report survey questions were analyzed by using descriptive statistics, such as means and SDs. For the semistructured interviews, data were analyzed by using a content-analysis 2-phase process (29). The first phase involved reviewing the notes to eliminate material not relevant to the study. The remaining relevant responses were then organized under 6 headings corresponding to the 3 questions about both home and work. The themes were reviewed by the research team as a validity check.

## Results

The ANCOVA conducted on the sample of participants who provided measures at baseline, postworkshop, and 6-week follow-up (n = 323) indicated significant effects for Time for 3 of the 4 outcome measures: antifat attitudes (*F*[1, 131] = 5.91, *P* = .02), internalization of media stereotypes (*F*[1,316]= 5.63, *P* = .018); self-efficacy to address weight bias (*F*[1, 317] = 43.90, *P *< .001). For body satisfaction, the overall effect for time was not significant (*F*[1,321] = 1.38, *P* = .22). The mean antifat attitude score was significantly lower after the workshop than at baseline (*P* < .001); the mean score by the 6-week follow-up period was significantly higher than the score for the period after the workshop (*P* = .02) (Table). However, the antifat attitude score remained significantly lower than at baseline (*P* < .001). The same pattern was seen with the mean scores for internalization of media stereotypes. The mean score at postintervention was significantly lower than at baseline (*P* < .001); by the 6-week follow-up, the score was significantly higher (*P* = .02) compared with postintervention but still significantly lower than the mean at baseline (*P* < .001). For the self-efficacy to address weight bias scores, the mean was significantly higher at postintervention than at baseline (*P* < .001). By the 6-week follow-up, the score was significantly lower than postintervention but remained significantly higher than at baseline (*P* < .001).

**Table:** Survey Scores to Assess Public Health Promoters’ Weight Bias and Eating Attitudes by Outcome Variable, Professional Development Pilot Study, Ontario, Canada, 2010-2011

Outcome Variable	Preintervention Mean Score (SD)	Postintervention Mean Score (SD)	6 Weeks Postintervention Mean Score (SD)
Antifat attitudes^a^	33.83 (16.83)	23.99 (14.04)	25.18 (15.02)
Internalization of media stereotypes^b^	13.61 (4.89)	11.48 (4.72)	12.28 (4.99)
Self-efficacy to address weight bias^c^	22.05 (2.63)	23.67 (2.64)	22.98 (2.53)
Body satisfaction^d^	19.65 (4.70)	21.00 (4.45)	20.58 (5.13)

Abbreviation: SD, standard deviation.

a Antifat attitudes assessed using the Antifat Attitudes Questionnaire (15) (possible scores ranged from 0 to 117). See Methods section for a description of how scores were determined.

b Internalization of media stereotypes assessed using the Sociocultural Attitudes Towards Appearance Questionnaire (16) (possible scores ranged from 5 to 25). See Methods section for a description of how scores were determined.

c Self-efficacy to address weight bias assessed using the Self-Efficacy to Change questionnaire (18) (possible scores ranged from 7 to 28). See Methods section for a description of how scores were determined.

d Body satisfaction assessed using the Body Satisfaction Scale (17) (possible scores ranged from 6 to 30). See Methods section for a description of how scores were determined.

### Intent to apply newly gained knowledge

Participants reported that they planned to reflect on their own personal biases about food and weight, integrate mental health promotion into their daily practice, and re-examine their workplace resources and revise them accordingly on the basis of what they learned in the workshop. Overall, they were eager to enhance their role-modeling skills, collaborate across different disciplines, and advocate for greater awareness and consideration of weight bias awareness and mental health promotion in their public health planning. A majority (61%) saw value in having follow-up sessions to help them synthesize the research that backed up the workshop information and to role-play ways to improve their delivery of health promotion messaging.

### Program satisfaction and feasibility

Most participants rated the workshop material as “excellent” and found it to be “credible, current, and evidence-based.” Their favorite parts were the research and information that was shared, the multimedia component, the opportunity for exchange and interaction, the presenters, the case studies that were presented, and the opportunity for reflection. Participants indicated they would have preferred spending more time engaging in practicing skill-building activities with the facilitators such as learning practical tips to avoid transmitting weight bias in their healthy weight messaging and learning how to reframe their language around weight. A majority of participants (69%) reported no discomfort in filling out the survey questions. Some reported that the survey questions helped them to reflect on their own values. A majority (78%) said that taking a full day to attend the workshop was feasible and that the length of the training was adequate (63%). A few stated a preference for the training to be extended to 2 or 3 days.

### Semistructured interviews conducted at the 6-week follow-up period

The content analyses of the follow-up interviews conducted with the randomly selected subset of participants (N = 42) indicated 4 overall themes: 1) heightened awareness of weight talk taking place at home or work, 2) heightened reflection on one’s own personal antifat attitudes, 3) feeling empowered by the newly gained knowledge that obesity prevention requires a call to action beyond the individual level, and 4) recognition of the need for additional mentoring support, to help them integrate their new knowledge into daily practice, and organizational support, to help them reframe their obesity prevention work by focusing on mental health promotion and social determinants of health.

## Discussion

This pilot study provides preliminary findings on a professional development intervention designed to decrease antifat attitudes and increase personal sense of efficacy to address weight stigma among public health workers engaged in health promotion. The role of these public health workers is to provide advice to the public. They work in partnership with community organizations and with varied groups, including children, youth, parents, families of young children, students and teachers in school boards, and adults in community and workplace settings. The training emphasized fostering a delivery style among the health promoters that balanced overall health, above and beyond weight, with the goal of minimizing the potential for unintended harm (ie, avoiding an overemphasis on weight and body mass index as physical parameters of health, or the moralizing of eating, that is, labeling foods as “good” or “bad”). These messages have been previously shown to trigger weight and shape preoccupation or unhealthy eating practices among children and youth (30). Finally, ways to integrate mental health promotion and resiliency-building into daily practice at the individual and community levels were introduced.

The findings indicated significant decreases among the public health participants in antifat (weight bias) attitudes and internalization of media stereotypes and significant increases in sense of self-efficacy to address weight bias. These intervention effects started to drift by the 6-week follow-up period, suggesting the need for “booster” sessions, or ongoing support. By the end of the intervention, participants reported greater understanding of the importance of considering socioecological factors other than just nutrition and physical activity when promoting wellness at the individual and community levels. Most participants indicated that they needed additional support practicing what they learned during the workshop. Critical incidents reported by participants after the workshop can provide a basis for decisions about future programming. Research is under way to determine whether postworkshop booster sessions help sustain the initial benefits brought on by the LENS intervention.

Our study has limitations. We did not have a control group, which prevents comment about the efficacy of the intervention, a goal for future studies. Also unknown is whether the study findings would be similar if facilitators came from different professional disciplines. Nevertheless, our findings indicate that a focus on weight bias awareness helps to reduce antifat attitudes, which, in turn, helps to promote access and equity, the cornerstone of public health core values (12). Increasing health promoters’ understanding of how mental health promotion can help them meet their target goals for promoting healthy lifestyles among the general public, and determining whether this approach has a direct influence on the public’s behaviors, deserves further study.

**Box. Three Themes of the Professional Development Workshop for Health Promoters**

1. Weight Bias Awareness
  - Reflection about personal beliefs about the causes of obesity, the source of those beliefs, the influence of one’s own upbringing on those beliefs, and one’s own prejudice toward overweight/obese people and how these factors influence daily work practice
  - knowledge translation of the research literature on the negative consequences of weight bias on both adults and children, in particular its negative influence on mental health and the uptake of health-promoting behaviors) (21–24)
2. A balanced approach to healthy eating and healthy weight messaging
  - Intuitive eating
  - Flexible approach to healthy eating
  - Early warning signs of disordered eating and excessive exercise
  - Aspects of obesogenic environments that influence eating and activity levels
  - Potential negative consequences of focusing exclusively on weight instead of overall health during the delivery of obesity prevention messaging
    - The triggering of food and weight preoccupation
    - Repeated cycles of weight loss and regain
    - Distraction from other personal health goals and wider health determinants
    - Reduced self-esteem
    - Disordered eating and other health problems
    - Weight stigmatization and discrimination
3. Mental health promotion
  - The association between mental and physical health
  - Reflection about one’s own personal style of coping
  - Self-assessment of stress management skills including assertive communication, linkages between the experience of stress and engagement in healthy lifestyles
  - Knowledge translation of the research literature on resiliency building as a strategy for the prevention of smoking, mood and depression, and disordered eating in youth
