# Erratum, Vol. 10, April 4 Release

**DOI:** 10.5888/pcd10.120185e

**Published:** 2013-04-18

In the article “Integrating Weight Bias Awareness and Mental Health Promotion Into Obesity Prevention Delivery: A Public Health Pilot Study,” an item in boxed text “Three Themes of the Professional Development Workshop for Health Promoters” was incorrect. The third theme, mental health promotion, should have listed only the following parts:

a. The association between mental and physical health

b. Reflection about one’s own personal style of coping

c. Self-assessment of stress management skills including assertive communication, linkages between the experience of stress and engagement in healthy lifestyles

d. Knowledge translation of the research literature on resiliency building as a strategy for the prevention of smoking, mood and depression, and disordered eating in youth

The correction was made to our website on April 18, 2013, and appears online at http://www.cdc.gov/pcd/issues/2013/apr/12_0185e.htm. We regret any confusion or inconvenience this error may have caused.

